# Supplementary figures and images for: High diversity and suggested endemicity of culturable Actinobacteria in an extremely oligotrophic desert oasis
Source: PeerJ. 2017 May 2;5:e3247. doi: 10.7717/peerj.3247 (PMC5417069; doi:10.7717/peerj.3247)

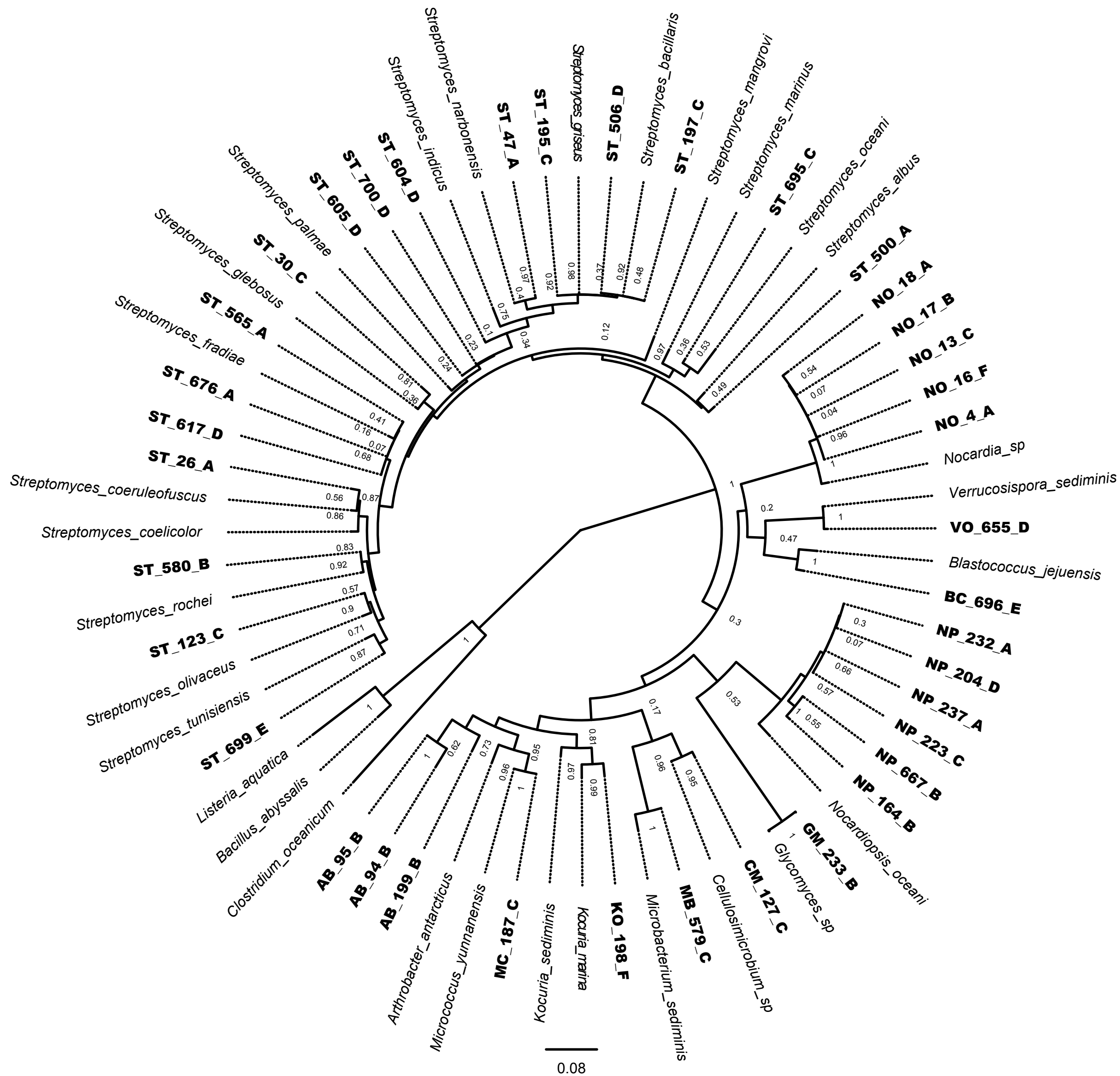

Supplement: Figure S1 — Phylum Actinobacteria Phylogenetic tree based on 16S rRNA sequences using Maximum Likelihood (ML) algorithm for representative CCB-isolated Actinobacteria and their closely related type strains. Bootstrap values based on 1000 replicates. [file peerj-05-3247-s002.pdf]
