# Supplementary material for: High diversity and suggested endemicity of culturable Actinobacteria in an extremely oligotrophic desert oasis
Source: PeerJ. 2017 May 2;5:e3247. doi: 10.7717/peerj.3247 (PMC5417069; doi:10.7717/peerj.3247)

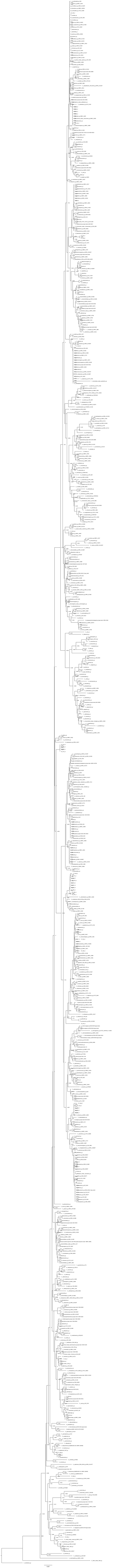

Supplement: Figure S2 — Phylogenetic tree based on a data set of 667 16S rRNA sequences of the Streptomycetaceae family using the neighbour-joining method. Bootstrap values based on 1000 replicates. [file peerj-05-3247-s003.pdf]
